# The CORE study—An adapted mental health experience codesign intervention to improve psychosocial recovery for people with severe mental illness: A stepped wedge cluster randomized‐controlled trial

**DOI:** 10.1111/hex.13334

**Published:** 2021-08-04

**Authors:** Victoria J. Palmer, Patty Chondros, John Furler, Helen Herrman, David Pierce, Kali Godbee, Konstancja Densley, Jane M. Gunn

**Affiliations:** ^1^ The Department of General Practice, Faculty of Medicine, Dentistry and Health Sciences, Melbourne Medical School The University of Melbourne Parkville Victoria Australia; ^2^ The ALIVE National Centre for Mental Health Research Translation The University of Melbourne Parkville Victoria Australia; ^3^ Orygen, The National Centre of Excellence in Youth Mental Health The University of Melbourne Parkville Victoria Australia; ^4^ Department of Rural Health The University of Melbourne Ballarat Victoria Australia

**Keywords:** codesign, community mental health services, experience‐based codesign, psychosocial recovery, quality improvement, severe mental illness, stepped wedge cluster randomized‐controlled trial

## Abstract

**Background:**

Mental health policies outline the need for codesign of services and quality improvement in partnership with service users and staff (and sometimes carers), and yet, evidence of systematic implementation and the impacts on healthcare outcomes is limited.

**Objective:**

The aim of this study was to test whether an adapted mental health experience codesign intervention to improve recovery‐orientation of services led to greater psychosocial recovery outcomes for service users.

**Design:**

A stepped wedge cluster randomized‐controlled trial was conducted.

**Setting and Participants:**

Four Mental Health Community Support Services providers, 287 people living with severe mental illnesses, 61 carers and 120 staff were recruited across Victoria, Australia.

**Main Outcome Measures:**

The 24‐item Revised Recovery Assessment Scale (RAS‐R) measured individual psychosocial recovery.

**Results:**

A total of 841 observations were completed with 287 service users. The intention‐to‐treat analysis found RAS‐R scores to be similar between the intervention (mean = 84.7, SD= 15.6) and control (mean = 86.5, SD= 15.3) phases; the adjusted estimated difference in the mean RAS‐R score was −1.70 (95% confidence interval: −3.81 to 0.40; *p* = .11).

**Discussion:**

This first trial of an adapted mental health experience codesign intervention for psychosocial recovery outcomes found no difference between the intervention and control arms.

**Conclusions:**

More attention to the conditions that are required for eight essential mechanisms of change to support codesign processes and implementation is needed.

**Patient and Public Involvement:**

The State consumer (Victorian Mental Illness Awareness Council) and carer peak bodies (Tandem representing mental health carers) codeveloped the intervention. The adapted intervention was facilitated by coinvestigators with lived‐experiences who were coauthors for the trial and process evaluation protocols, the engagement model and explanatory model of change for the trial.

## 1 INTRODUCTION

Internationally, mental healthcare policies are replete with references to embed coproduction and codesign with service users in the design, planning and delivery of programmes.^1^, ^2^, ^3^, ^4^, ^5^, ^6^, ^7^, ^8^, ^9^ The 2017 United Nations Special Report identified coproduction as fundamental to mental health service participation to reach the highest attainment of physical and mental health.^10^ This is coupled with consensus for recovery‐oriented mental health services that consistently facilitate psychosocial recovery as a subjective, ongoing process that encompasses spiritual, social, psychological and cultural dimensions for individuals.^11^, ^12^, ^13^ Engaging mental health service users is central to the enactment of recovery‐oriented systems and to ensure that participation in service design, planning and delivery holds personal and social meaning for individuals. Despite a growing evidence base that supports an association between engagement leading to improved patient experience, clinical effectiveness and patient safety,^14^ a more recent review of service user participation in mental healthcare planning and programmes to improve experience and service effectiveness found that exclusion continued to be the norm rather than the exception.^15^ There is also cautiousness emerging about engagement as the next big blockbuster drug for healthcare and the driver for improved health outcomes, quality and safety and reduced healthcare costs.^16^, ^17^

In the attempts to implement systematic approaches to engage people who access mental healthcare services and to ensure that the engagement methods do foster shared power and decision‐making, interest has grown exponentially in participatory methods such as codesign and coproduction. In the last decade, a rapid evolution of studies labelled as codesign, coproduction, coinnovation and cocreation has occurred in healthcare quality improvement. This evolution has contributed to what has been called a Participatory Zeitgeist, where participation using codesign and coproduction has become the spirit of our contemporary times, but not without conceptual and definitional challenges and a need for robust evaluation.^18^ For example, the extent to which engagement using codesign leads to recovery‐oriented service delivery, individual empowerment or improved health outcomes in mental health services is yet to be determined.

One quality improvement, participatory method to engage service users, carers and staff in service design where experience is central is experience‐based codesign (EBCD). EBCD aims to improve experiences of services by working in partnership with staff, service users and carers on areas for change.^19^, ^20^ EBCD has been implemented to improve service experiences and outcomes in head, neck, breast and lung cancer services, gynaecology and colorectal settings, stroke and rehabilitation, emergency, to end of life and intensive care units and to a much lesser extent in mental health.^21^ While organisational improvements such as operational efficiencies, interpersonal dynamics of care, increased communication, team relationships, patients feeling listened to and reduced complaints have been documented,^22^, ^23^ no studies have examined the impact of codesigned improvements on systems or service levels or on individual health outcomes. No randomized‐controlled trial (RCT) study designs have been used to date to test this.

To address this gap and to identify the benefits or otherwise of an adapted EBCD method for recovery‐oriented mental health services and improved psychosocial recovery of service users, the CORE study tested the effectiveness of mental health experience codesign (MH ECO). A stepped wedged cluster randomized‐controlled trial (SW‐CRT) was conducted in nonclinical (psychosocial recovery‐oriented) Mental Health Community Support Services (MHCSS) in Australia. CONSORT guidance for reporting SW‐CRT was followed.^24^ Our primary participants were people living with severe mental illness (SMI was defined as including psychosis, schizophrenia, bipolar disorder, major depression and other disorders such as personality and eating disorders), with carers who had a family member engaged in services and staff.^25^ The secondary outcomes were improved quality of life for people living with SMI and carers, and changes to recovery attitudes from staff and the recovery‐orientation of services. This paper reports on the trial outcomes for people with SMI only; carer and staff outcomes are reported separately.^26^

## 2 METHODS

### 2.1 Trial design

A SW‐CRT was conducted between 2013 and 2017 using an open cohort design (meaning both cross‐sectional and longitudinal data were included).^27^ Trial registration was completed with ANZCTR (No. 12614000457640) before recruitment commenced; the study protocol and a statistical analysis plan were published (2015; May 2017) before the final follow‐up period of data collection was completed.^28^ The intervention, MH ECO (explained below), was directed at the service level for improvements, so a cluster design was determined to be the most appropriate. Four large mental health organisations (two nongovernment providers and two community health centres) were partners in the trial and the intervention was delivered to nine teams across these four organisations in metropolitan, outer metropolitan and regional locations. Teams were randomly allocated (three at a time) to different start dates 9 months apart (see Table 1).

**Table 1:** Schematic of stepped wedge cluster randomized trial for the CORE study

	Follow‐up time			
Arms	Wave 0	Wave 1	Wave 2	Wave 3
	Baseline	9 months	18 months	27 months
1	0	9 months	18 months	27 months
2	0	0	9 months	18 months
3	0	0	0	9 months

*Note:* 0 = control phase; 9, 18, 27 months = intervention phases (indicates the length of time since the start of the intervention).

Arm is the allocation of group of clusters/individuals. Three clusters were randomized to each arm.

## 3 PARTICIPANTS

### 3.1 Settings

Nine teams delivering psychosocial recovery programmes as part of commissioned MHCSS were recruited across four large service providers in Victoria, Australia. Organisations had delivered services to approximately 14,000 people in any given year at the time of recruitment in 2013. Support included daily living skills development, recovery planning and facilitation of social and community participation to people living with SMI in community settings. At the time of trial commencement, an outreach model of individual support was implemented shifting away from on‐site, group models of service. The goal of MHCSS programmes is to support psychosocial recovery and deliver recovery‐oriented mental healthcare.

The most available data on MHCSS service recipients showed people lived with between one and four complex factors, which included social isolation, activities of daily living, issues related to unresolved trauma, treatment‐resistant symptoms, extensive time to maintain levels of functionality with little improvement in functionality over time, chronic physical health problems, difficulty maintaining medications, problems with intellectual disability/cognition, alcohol use and drug use. MHCSS staff were not responsible for clinical assessments, though they engaged with clinical care providers for updates and information sharing.

### 3.2 Service recipients

Eligibility for people living with SMI to be recruited to the study followed service eligibility criteria set by the government funder. MHCSS service users were characterized as having enduring psychosocial disabilities and long‐term impairments from mental illnesses that range from diagnostic names such as bipolar disorder, schizophrenia, psychosis, chronic depression and anxiety to obsessive compulsive disorders and other personality disorders. Inclusion criteria to services were as follows: aged between 16 and 75 years and a psychiatric condition (bipolar disorder, schizophrenia, psychosis, major depression, severe anxiety, personality disorder, posttraumatic stress) that results in persistent impairment and substantial reduction in psychosocial functioning for communication, social interaction, learning, self‐care and self‐management affecting social and economic participation. Carers were eligible for recruitment to the study if they were a family member, friend or in a caring relationship with the person living with SMI; carers did not need to be matched. People with SMI and carers were not recruited if they could not understand spoken English and were unable to complete the two‐stage consent process (outlined in the published protocol) or were not in receipt of services from a participating team. The rationale for exclusion of people who could not understand spoken English was due to the primary and secondary outcome measures not being appropriately translated for the cultural communities in question, interpreter availability and the challenges presented by multiple languages with an interpreter within the face‐to‐face codesign sessions.

### 3.3 Intervention

**Figure 1:**
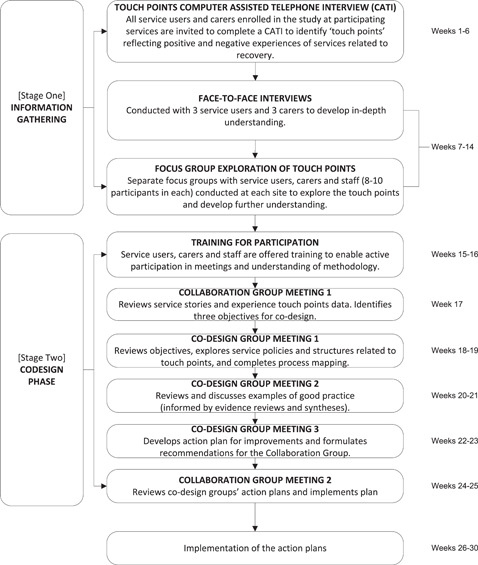
Adapted mental health experience codesign intervention

The adapted MH ECO intervention^18^, ^28^ is described in the published trial and nested process evaluation protocols.^28^ The MH ECO model was codeveloped by the state consumer (Victorian Mental Illness Awareness Council) and state carer (Tandem representing Victorian Mental Health Carers) peak agencies in Victoria, Australia, in partnership with the Victorian State Government. The model was piloted and evaluated in Psychiatric Disability and Rehabilitation Support Services (now called MHCSS) before this trial and a short explanatory video was produced. Two lived‐experience coinvestigators (a consumer and a carer) participated in intervention adaptations, and delivered training for codesign preparation and all codesign meetings. Figure 1 illustrates the adapted MH ECO intervention for the trial in two stages: information gathering (Stage 1) and codesign (Stage 2).

To implement Stage 1, positive and negative touch points were identified per cluster (e.g., per individual service teams) over 6 weeks. University‐based telephone interviewers received training from each lived‐experience coinvestigator about working with people living with SMI and carers. Service users and carers were asked to share stories about: ‘a time when something went well’; ‘a time when something could have gone better’ and ‘the things that stood out about those experiences’; and ‘how ideal care might look’ (this included prompts about involvement in decision‐making and being informed about services). The above three open‐ended interview questions were used instead of the longer survey version originally planned as the team sought to maintain the narrative and qualitative focus underpinning EBCD. The film component usually employed within EBCD to share experiences with staff was not used as the service partners indicated that this was not a preferred option for people from their previous experience.

Responses to telephone interview questions were analysed by group using the Leximancer software analytics programme. Leximancer organizes prominent concepts discussed in text within themes into a thematic map.^29^ Thematic maps were reviewed to identify the most commonly shared negative touch points per group and textual responses examined to understand nuanced meanings. These touch points were then explored in focus groups with service users, carers and staff (held separately) as neutral statements to develop a deeper understanding of people's experiences. Emotion mapping was completed using brighter colour post‐it notes to represent strong feelings and pale colours to represent less strong/mild feelings (the written feedback that participants provided could be either positively or negatively framed; the focus was on emotional connection to the touch point).

**Figure 2:**
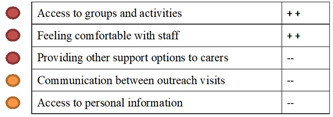
Illustration of emotion mapping and synthesis of service stories for presentation to collaboration groups to formulate codesign objectives

Emotion mapping and how this was used to identify shared patterns across groups is presented in Supporting Information Appendix 1. Once the shared touch points for improvement areas were determined in a cluster, a summary of the service stories was provided to the Collaboration Group (detailed in Figure 2) as a short report for development of the codesign objective.

All participants received training before codesign meetings (6 h over 2 half days). Training included activities to explore previous experiences of working in groups and with staff and ways to determine power dynamics; it encouraged people to be open, to listen together, to foster comfort in meeting with others, build confidence and set the parameters for respectful ways of working. The short video from the pilot was played for participants to share the MH ECO approach and the experiences that other participants had shared. Staff joined training after the service user and carers completed their sessions. This was to ensure adequate time to explore any negative experiences of previous group work and to address concerns around power dynamics in working with staff.

Codesign groups worked with the codesign improvement objectives set out by the collaboration group over three facilitated meetings (2 h each). They codesigned (a) a process map in relation to the codesign objective. The process map led to the identification of sticking points related to the area for improvement and this supported narrowing the focus of what might need to be codesigned for changes to be implemented. (b) They brainstormed improvements to implement. The research team provided a brief evidence synthesis of any initiatives related to a codesign objective to inform the codesign of improvements. (c) Solutions were formulated for implementation and these were presented in an action plan. The final part of Stage 2 involved the collaboration group reviewing the action plan and cocreating an implementation plan for the service. A research team member completed implementation check‐ins with staff to collect data on the barriers and enablers in services for implementation of the codesigned improvements. Clusters that were in the control phase received a questionnaire to check on staff numbers to ensure balance across groups and reduce the possibility of contamination. The implementation check‐ins will be reported separately in the nested process evaluation paper for the trial. All participants were invited to complete an open‐ended feedback form for training and codesign sessions. A summary of the feedback from all participant groups can be found in Supporting Information Appendix 2.

### 3.4 Outcomes

Trained telephone interviewers collected data at the cluster and individual levels at baseline (October 2014–July 2015) before randomisation and subsequently at 9 (January–February 2016), 18 (October–November 2016) and 27 (June–July 2017) months postrandomisation. The trial was approved by the University research ethics committee, registered with ANZCTR (No. 12614000457640) and conducted in accordance with the published trial protocol with only changes to the telephone interview.

Participants were asked to complete a structured questionnaire that included the 24‐item Revised Recovery Assessment Scale (RAS‐R) as the primary outcome measure.^30^ The measure for psychosocial recovery for service users was identified in a small pilot completed with 40 people who were recruited from the partner agency VMIAC. Service users completed combinations of either the 24‐item RAS‐R (*N* = 20), the 26‐item Maryland Assessment of Recovery in People With Serious Mental Illness (*N* = 17) or the RAS‐R and person in recovery version of the 36‐item Recovery Self‐Assessment Scale (*N* = 13). Measures were completed in written form and/or over the telephone by different groups for acceptability and feasibility. There was overwhelming positive feedback for the RAS‐R.

The RAS‐R uses a 5‐point Likert rating scale for each item, from 1 = ‘Strongly Disagree’ to 5 = ‘Strongly Agree’; scores range from 24 to 120, and higher scores indicate greater recovery. There are five RAS‐R subdomains: (i) personal confidence and hope (nine items; range: 9–45), (ii) willingness to ask for help (three items; range: 3–15), (iii) goal and success orientation (five items; range: 5–25), (iv) reliance on others (four items; range: 4–20) and (v) no domination by symptoms (three items; range: 3–15). Higher ratings within domains indicate greater recovery.^30^ The secondary outcome quality of life was measured using the shortened eight‐item version of the World Health Organisation Quality of Life Scale (EUROHIS‐QoL eight‐item index).^31^ EUROHIS‐QOL measures personal satisfaction on eight different aspects of life: overall quality of life, general health, energy, daily life activities, self‐esteem, relationships, finances and home. Each item is scored on a five‐point Likert scale ranging from 1 = ‘Not at all’ to 5 = ‘Completely’; score range is between 8 and 40, and higher scores indicate better quality of life. EUROHIS‐QOL was selected because recovery‐oriented mental health services ideally should improve general health, daily life activities, self‐esteem, relationships, financial and home life.

Participants were asked additional questions about previous hospitalisations, any previous involvement in service improvement activities with service providers, physical health conditions in the last 12 months, physical activities and perceived challenges that individuals felt they faced in the next 12 months. A subsample of participants consented to qualitative data collection that included sharing a timeline of times of being well or unwell, a week‐in‐the‐life diary and a social network map. In addition to evaluation feedback at the end of every collaboration or codesign meeting, a subsample of participants (service users, carers and staff) was interviewed for the nested process evaluation. These interviews were conducted face‐to‐face and/or by phone; the analysis of these data will be presented separately in the nested process evaluation for the trial.

### 3.5 Sample size

Sample size was powered for at least 80% to detect a standardized effect size of 0.35 for psychosocial recovery RAS‐R (primary outcome) between the intervention and control phases for a fixed cluster size of 30 people with SMI from nine clusters at each of the four follow‐up times (see Table 2 in the full published study protocol).^28^ Power was determined in a simulation study that assumed an intracluster correlation of 0.1, a 5% alpha level for a two‐sided test, different probabilities that each individual would remain in the cluster at each follow‐up time point (0, 20% and 60%) and within‐individual correlations of 0.2 and 0.7 for service users that contributed observations to two or more consecutive follow‐up time points (reported in the published study protocol). Interim analyses and stopping rules were not required.

**Table 2:** Trial participant flow

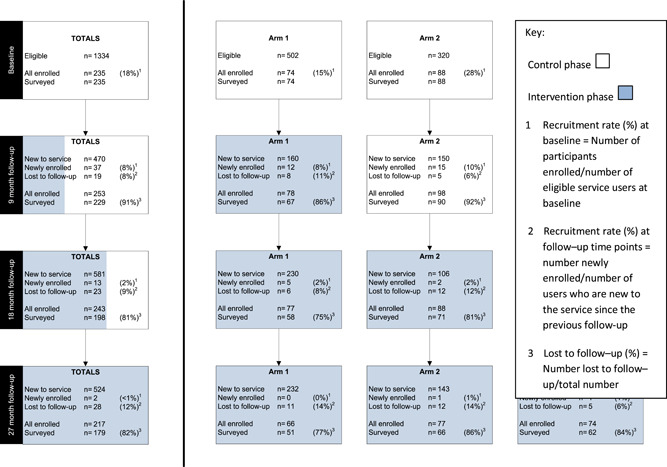

*Note*: At baseline, 1334 invitation letters were delivered to the service organisations for each of the nine clusters. Thirty‐seven were returned to the sender. It was not possible to track whether services sent all the letters or whether all letters reached intended recipients due to ethics requirements.

### 3.6 Randomisation

A computer‐generated random allocation sequence stratified by the MHCSS organisation was generated by the statistician blinded to cluster identity and not involved in assessment or intervention delivery. The trial coordinator received the order for delivering the intervention. Study participants, facilitators, telephone researchers and staff assisting at intervention meetings were blinded to the allocation sequence during recruitment and baseline data collection. All participants provided audio‐recorded consent. Once assigned to the intervention, participants could no longer be blinded to their status due to the face‐to‐face components of the intervention. Participants in the control phases at Wave 1 were blinded to whether they would receive the intervention during the second or third wave. Clusters allocated to the Wave 1 intervention phase were notified after baseline data were completed. Participants in Waves 2 and 3 were informed of intervention commencement at the start of their allocated waves. Research interviewers collecting outcome data remained blinded to the intervention status of the participants.

### 3.7 Statistical methods

Descriptive statistics were used to summarize the characteristics of people living with SMI on first entry to the open cohort (either at baseline, 9, 18 or 27 months) by arm and for the entire sample. A linear mixed‐effects model compared the intervention and control phases for each continuous outcome. Each outcome measured at each follow‐up time was arranged into a single variable, and a second variable was created that identified the time point at which the data were collected. The model included indicator variables for the study arm (0 = control phase, 1 = intervention phase) and the follow‐up time (1 = baseline, 2 = 9 months, 3 = 18 months, 4 = 27 months) as fixed effects. The intercept was constrained to be equal during the control phases because we expected no intervention effect. Cluster and individuals were treated as random effects to account for the correlation of outcomes of individuals who belonged to the same cluster (within‐cluster correlation) and repeated measures on the same individual over time (within‐individual correlation).^32^ Estimates of the intervention effect were reported as a difference in the mean outcome between the intervention and control phases, with respective 95% confidence intervals (CIs) and *p* values. For these analyses, the underlying assumption was that treatment effect was constant across the different individuals (both cross‐sectional and longitudinally) and at the different time points, regardless of length and level of exposure to the intervention phase. In a prespecified secondary analysis, the estimates of the intervention effect were adjusted for education level, employment status and quality of life measured at baseline.^31^ Analyses were conducted using Stata statistical software 13.1.^33^

An intention‐to‐treat approach analysed all study participants according to the arm that the cluster was assigned to at each time point.^34^ People who refused or were unable to complete follow‐up questionnaires were asked to complete the primary outcome measure to minimize missing outcome data. Up to five attempts were made over the 2‐month data collection period for outcome measures. This included contacting trusted proxies who were provided by individuals at enrolment. The reasons for why individuals were lost to follow‐up were recorded. Under the mixed‐effects models used for the analysis, data were implicitly assumed to be missing at random.^34^

In a planned secondary analysis, direct effects of the length of time for which the participants were in the intervention phase (namely, 0, 9, 18 and 27 months) at each follow‐up time were estimated using a linear mixed‐effects model, where the length of time exposed to the intervention was treated as a fixed effect and adjusted for follow‐up time. Estimates were reported as the mean outcome difference when exposed to the intervention for 9, 18 or 27 months compared to ‘0 months’, the time when individuals were in the control phase and not exposed to the intervention. The log‐likelihood ratio test was used to assess whether to treat the length of time in the intervention phase as a continuous rather than a categorical variable in the regression model. Treating exposure time to the intervention as continuous assumed that the increase in the intervention effect was linear with the length of time in the intervention phase. In sensitivity analyses, long‐term intervention exposure was investigated using the same methods as above, but restricted to participants recruited at baseline only.

## 4 RESULTS

Table 2 shows the participant flow of people with SMI in each arm at each follow‐up time. In total, 287 people with SMI (91 in Arm 1, 106 in Arm 2 and 90 in Arm 3) enroled in the study and contributed to at least one follow‐up time point. Of these, 235 (81.9%) were recruited at baseline, 37 (12.9%) at 9 months, 13 (4.5%) at 18 months and 2 (0.7%) at 27 months. Of the 52 recruited at subsequent time points, 31 people with SMI (59.6%) were from clusters that had not yet received the intervention.

A total of 841 observations were completed with 287 participants (an average of 2.9 observations for each participant) recruited from within the nine clusters (average 93.4 observations per cluster, with a range between 52 and 123). More than half of the participants who enroled at baseline contributed observations to four‐time points (130/235, 55%), 14% (34/235) to three‐time points, 16% (37/235) to two‐time points and the remaining 15% (34/235) to baseline only (see Supporting Information Appendix 3).

The mean of the RAS‐R scores was similar between the intervention (mean = 84.7, SD= 15.6) and control (mean = 86.5, SD= 15.3) phases. This was observed in the RAS‐R subdomains and the EUROHIS‐QOL index (see Supporting Information Appendix 4). Estimated within‐cluster and within‐individual correlations for the total RAS‐R score were 0.02 and 0.73, respectively. Supporting Information Appendix 5 provides a table that shows the within‐cluster and within‐individual correlations for the primary and secondary outcomes, estimated using the linear mixed‐effects model in the primary analyses.

Of the 37 new people with SMI enroled at 9 months, 22 (59%) contributed outcome data to all three subsequent time points, 6 (16%) to two‐time points and the remaining 9 (24%) completed only one survey. Nine (69%) of the 13 individuals recruited at 18 months contributed data at 27 months. Overall, 83% (238/287) of the people with SMI contributed at least two observations. Twenty‐four percent (70/287) were lost to follow‐up: 27% (25/91) in Arm 1; 27% (29/106) in Arm 2; and 18% (16/90) in Arm 3. Acute illness and no further interest in taking part were the two most common reasons cited for withdrawal.

Table 3 summarizes the demographic, clinical characteristics and carer relationships at baseline for people living with SMI. Overall, the characteristics of the individuals enroled at the baseline were balanced between the three study arms. The characteristics of the 52 individuals who enroled in the trial after baseline were also similar across the three arms (results not shown), although they tended to be slightly younger on average than the people with lived‐experiences of SMI recruited at baseline and they had a shorter duration of psychiatric illness. This may reflect that individuals recruited at baseline before randomisation included people who had been with the service for a longer period. They might have been older compared to those who were enroled at subsequent time points or for those who may have been new to the services.

**Table 3:** Characteristics of people with SMI enroled at baseline and postbaseline (9, 18 and 27 months) (*N *= 287)

	People with SMI enroled at baseline (*N* = 235)				People with SMI enroled postbaseline (*N* = 52)
	**Total **(*n* = 235), mean (SD)	**Arm 1 **(*n* = 74), mean (SD)	**Arm 2** (*n* = 88), mean (SD)	**Arm 3, **(*n* = 73) mean (SD)	**Total **(*n* = 52), mean (SD)
Age (years)	50.5 (12.7)	48.1 (14.0)	52.0 (12.2)	50.9 (11.7)	44.7 (12.5)
RAS‐R score (range: 24–120)	87.1 (15.3)	84.6 (17.0)	89.7 (15.3)	86.5 (13.1)	–
EUROHIS‐8 QoL index (range: 8–40)	25.6 (6.6)	24.0 (6.7)	27.3 (6.5)	25.1 (6.3)	–
Duration of longest‐standing mental health condition (years)^a^	18.1 (12.7)	17.6 (14.0)	17.0 (11.7)	19.9 (12.6)	14.6 (13.1)
Age at first hospital admission (years)^b^	32.3 (13.1)	34.6 (14.1)	33.5 (13.1)	28.7 (11.5)	31.9 (12.6)
Duration of the caring relationship (years)^c^	11.6 (13.9)	9.9 (12.9)	9.8 (12.3)	14.9 (15.9)	10.9 (15.2)
	***n* (%)**	***n* (%)**	***n* (%)**	***n* (%)**	***n* (%)**
Female^d^	155 (67)	52 (71)	61 (69)	42 (58)	20 (58)
Born in Australia	199 (85)	67 (91)	73 (83)	59 (81)	41 (80)
English is the first language	222 (95)	72 (97)	83 (95)	67 (92)	48 (96)
Education (highest level)					
Left school before completing Year 10	43 (18)	13 (18)	18 (20)	12 (16)	8 (16)
Completed Year 10 or equivalent	62 (16)	21 (28)	26 (30)	15 (21)	15 (29)
Completed Year 12 or equivalent	28 (12)	13 (18)	9 (10)	6 (8)	9 (18)
Certificate or diploma	67 (29)	16 (22)	24 (27)	27 (37)	14 (27)
Bachelor degree or higher	35 (15)	11 (15)	11 (13)	13 (18)	5 (10)
Currently working^e^	48 (20)	17 (23)	19 (22)	12 (16)	10 (19)
Pension/benefit is the main source income^e^	213 (91)	66 (89)	77 (88)	70 (96)	46 (88)
Self‐reported physical health is a problem^f^	169 (76)	50 (77)	62 (72)	57 (80)	43 (83)
Number of self‐reported mental health conditions^g^					
None reported	12 (5)	3 (4)	7 (8)	2 (3)	1 (2)
1	62 (27)	18 (24)	21 (24)	23 (32)	16 (34)
2	71 (30)	21 (28)	28 (32)	22 (31)	15 (32)
3 or more	88 (38)	32 (43)	32 (36)	24 (34)	15 (32)
Self‐reported mental health conditions^g^, ^h^					
Major depression	121 (52)	39 (53)	47 (53)	35 (49)	25 (53)
Anxiety disorders (excluding posttraumatic stress disorder)	97 (42)	35 (47)	36 (41)	26 (37)	19 (40)
Schizophrenia and other psychotic disorders	85 (36)	27 (36)	28 (32)	30 (42)	12 (26)
Bipolar disorder	70 (30)	20 (27)	25 (28)	25 (35)	11 (23)
Personality disorders	39 (17)	19 (26)	12 (14)	8 (11)	9 (19)
Posttraumatic stress disorder	29 (12)	13 (18)	10 (11)	6 (8)	10 (21)
Substance use disorder	10 (4)	4 (5)	4 (5)	2 (3)	2 (14)
Eating disorders	6 (3)	3 (4)	3 (3)	0 (0)	1 (2)
Admitted to hospital for mental health	183 (78)	64 (86)	63 (72)	56 (77)	33 (63)
Has a carer	124 (53)	40 (54)	40 (45)	44 (60)	25 (48)
Currently living with carer^c^	63 (51)	16 (40)	25 (63)	22 (50)	11 (44)
Relationship to carer^c^					
Partner	24 (19)	7 (18)	7 (18)	10 (23)	6 (24)
Family member	71 (57)	26 (65)	23 (57)	22 (50)	14 (56)
Friend	13 (10)	3 (8)	4 (10)	6 (14)	2 (8)
Other	16 (13)	4 (10)	6 (15)	6 (14)	3 (12)

*Note:* Discrepancies in totals due to missing responses.

Abbreviation: RAS‐R, 24‐item Revised Recovery Assessment Scale; SD, standard deviation; SMI, severe mental illness.

^a^ Duration of any longest‐standing mental health condition self‐reported by participants. *N* = 206 at baseline (66 in Arm 1, 75 in Arm 2 and 65 in Arm 3) and 42 postbaseline participants.

^b^ Based on responses to the question, ‘How old were you the first time you were admitted to hospital to get help for your mental health?’ 216 service users (75%) who reported a hospital admission related to mental health were asked this question.

^c^ *n* = 149 (52%) participants who reported having a carer at the time of entry into the study.

^d^ One respondent in Arm 1 and one in Arm 3 selected ‘Rather not say’. They have been coded as missing for the analysis.

^e^ Based on responses (yes/no) to the questions, ‘Do you work?’ and ‘Is a pension or benefit your main source of income?’ at entry into the study.

^f^ Based on responses to the question, ‘In the past 12 months, have you had any problems with your physical health?’

^g^ Based on participant responses to the question, ‘Have you ever been given a name for your condition? If yes, what is the name?’. All mental health conditions reported were included in the total.

^h^ Self‐reported health conditions are not mutually exclusive. Eight self‐reported mental health conditions were selected and grouped into the ICD‐10 Classification for Mental and Behavioural Disorders Diagnostic Criteria for Research established by the World Health Organisation 1993.

Table 4 presents the negative touch points identified to inform service improvements and codesigned objectives across the nine clusters.

**Table 4:** Touch point category, themes for improvement and the implemented codesigned solutions from a mental health experience codesign intervention

Touch point broad service area of connection	All themes related to touch points for people living with severe mental illness and carers on what could be better	Codesigned solutions that were implemented within the funded period of the trial^a^
Continuity of care: Holistic care	Facilitate connection between services Provide colocated medical and nonmedical services Integrated support for people with multiple and complex needs Consistency of support workers, staff rescheduling of appointments Service user‐driven care Knowing a story and what is happening in someone's care—relating to someone as a person	Designed and implemented a secondary worker process for when staff were on leave
Social connection (groups)	Providing a variety of groups, flexible drop‐in options to connect with other people Localized group with common needs, geographically local groups Worker presence in social activities Shared life experiences with group members not just membership based on illness	Newsletter options developed to share information Designed and implemented calendar of events to distribute via email and or web Provided WiFi access to service users to increase internet use for information finding Developed a Facebook page for organisation and service user contact
Communication	Better communication between outreach visits Communication about service changes and models of case management and progress made Follow up with someone when they try to connect to a service	Technical change to the voicemail system and answer machine messages updated Outreach policies updated at service Websites reviewed and updated in some services
Service engagement	Opportunity to give feedback and be updated on feedback Feeling heard Feeling needs are heard by the organisation	Designed and implemented a feedback system in conjunction with the distribution of calendar of events Implemented feedback box near reception
Physical infrastructure	Feeling welcome at service	Redesigned receptionist space for more of a welcoming experience on arrival and waiting
Public and private information	Public: Information about what groups exist at a service Private: Access to private information and treatment records	More information provided on websites
Carers	Informing and involving carers with updates about services provided to a person they care for Communication with and involvement of carers in care planning and outcomes Support options to carers for when they are unwell themselves Communication about support groups for carers directly Time to process information when first engaging at a service	Implemented carer peer support workers within service delivery Provided information to staff on the role of carer workers Designed new brochure and website updated (involved carers in the design of these) Increased activities for carers and options for access to self‐care programmes

^a^ Not all touch points for improvement were addressed within the codesign stage of the intervention due to trial limitations to focus on one area.

Table 5 shows the estimated intervention effect comparing the intervention and control phases (primary analysis) and the direct effects of the length of exposure (duration) to the intervention, adjusted for the time point the outcome was measured. The adjusted estimated difference in the mean RAS‐R score between the intervention and control phases was −1.70 (95% CI: −3.81 to 0.40; *p* value = .11), with the CI excluding the minimum hypothesized difference of 5.4, given a standard deviation of 15.3 at baseline. There was no evidence to support time‐specific intervention effects. The results did not change after adjustment for confounders or when analyses were repeated for only the 235 participants enroled at the baseline cohort only (results not shown). Similarly, there was no evidence to support improvements in the RAS‐R subdomains of personal confidence and hope, willingness to ask for help, goal and success orientation, reliance on others, no domination by symptoms or in the EUROHIS‐QOL index between the intervention and control phases, or time‐specific intervention effects (Table 3). No significant harms or unintended effects were reported.

**Table 5:** Estimated intervention effect for the primary and secondary outcomes for people with SMI (*N* = 287,841 observations)

					Time‐specific intervention effect									** *p* Value**
**Outcomes**	Intervention vs. control phase				9 months			18 months			27 months			
	**Coeff** a	**95% CI**		***p* value**	**Coeff** ^**2**^	**95% CI**		**Coeff** b	**95% CI**		**Coeff** b	**95% CI**		
RAS‐R scores (range: 24–120)	−1.70	−3.81, 0.40		.11	−2.04	−4.30, 0.22		−2.22	−5.64, 1.21		−3.54	−8.65, 1.57		.36
RAS‐R subdomain scores														
Personal confidence and hope (range: 9–45)	−0.54	−1.47, 0.38		.25	−0.85	−1.83, 0.14		−1.48	−2.97, 0.01		−2.34	−4.57, −0.11		.21
Willingness to ask for help (range: 5–25)	−0.34	−0.80, 0.12		.14	−0.34	−0.82, 0.14		−0.22	−0.93, 0.50		−0.30	−1.36, 0.76		.50
Goal and success orientation (range: 3–25)	−0.32	−0.92, 0.27		.29	−0.48	−1.10, 0.15		−0.58	−1.52, 0.36		−1.23	−2.62, 0.17		.35
Reliance on others (range: 4–20)	−0.19	−0.67, 0.28		.43	−0.14	−0.64, 0.37		−0.10	−0.86, 0.65		0.12	−1.00, 1.25		.79
Not dominated by symptoms (range 3–15)	−0.32	−0.85, 0.22		.25	−0.28	−0.85, 0.29		0.09	−0.76, 0.95		0.03	−1.24, 1.30		.41
EUROHIS‐8 QoL index (range: 8–40)	−0.21	−1.17, 0.75		.67	−0.10	−1.13, 0.93		−0.16	−1.72, 1.40		0.36	−1.97, 2.69		.88

Abbreviations: CI, confidence interval; RAS‐R, 24‐item Revised Recovery Assessment Scale; SMI, severe mental illness.

^a^ Mean difference in outcome between the intervention and control phases (reference)

^b^ Mean difference in outcome between the length of time exposed to the intervention and being in the control phase (zero months is the reference).

CI, confidence interval

## 5 DISCUSSION

This is the first SW‐CRT designed trial to investigate whether a MH ECO intervention to increase recovery‐orientation of services would lead to increased psychosocial recovery outcomes and improved quality of life for people living with SMI. The primary outcome measure of psychosocial recovery was selected because the MH ECO pilot data had identified experiences of hope and empowerment and meaningful participation aligned with personal recovery definitions.^35^ These findings are also consistent with published qualitative evaluations of EBCD improvement projects. In those studies, participant involvement in EBCD has been described positively and there has been an emphasis on sharing stories as a practice in meaning‐making and participation in codesigned improvements as equal partners as generating hope.^21^

Many of the service improvements that resulted from the adapted MH ECO intervention centred on communication and information flow to and from services, the involvement of service users in programme design and delivery, and information about local activities and readiness to participate in groups. Service users also highlighted a desire to be able to meet people from similar life backgrounds, but not solely because they shared a diagnosis of a mental illness. Service users also described re‐design needs for physical spaces in services, and feeling unwelcome because of unfriendly or distant voicemail messages at services. In relation to this, service users wanted to see an increased use of SMS to receive appointment reminders, make changes to their own appointments or to receive information about staff absences.

These improvement areas can be understood within four categories that were initially identified in the seminal head and neck cancer centre EBCD projects conducted in the United Kingdom.^22^ The four categories were quick fixes—improvements that involved little or no change in everyday working practices (e.g., revising information, updating brochures); process redesigns—improvements where new in‐service procedures for consent or access to services were developed; cross‐service or interdisciplinary redesign—improvements involving process or structural redesign across different services to improve responses to an issue; and organisational change—improvements addressing organisational issues such as delayed receipt of results of a procedure or appointment wait times. Two further categories were added from this trial: Technological fixes—improvements to technological components of services such as websites, internet access, social media and app provision, phone and voicemails or SMS use, and physical infrastructure—improvements where there were changes to the physical environments of services.

Overall, the most prominent codesigned improvements implemented in the trial were related to quick fixes and process redesigns. New information and welcome packs were cocreated with service users, or information about social groups was gathered and presented on websites. A commonly reported barrier for uptake of the local information, though, was that service users tended to have limited use of emails and did not read the websites or typically use social media. The lessons here might be most relevant to future digital mental health transformation and the implementation of technology‐based interventions where purposive methods for engaging people will be needed.

In one or two services, attempts were made for organisational change and physical infrastructure improvements. Organisational changes that were implemented led to a coproduced camp by and for service users in one service, and in another service, a carer peer worker was arranged as a response to specific carer engagement needs. The implementation check‐ins conducted by the research team found that the carer peer worker role was not sustained, which does echo the published literature on recovery models, which suggests that the mere addition of peer workers within services and teams in isolation of changes to other parts of the service culture may be inadequate to foster recovery‐oriented services.^25^ In terms of physical infrastructure, one service redesigned a reception area to create a more welcoming environment and to display information brochures more prominently. Other codesigned improvements that resulted from the adapted MH ECO intervention included extra feedback mechanisms, the provision of internet access for service users and a coaching model to support readiness for community group participation.

Overall, we successfully implemented the MH ECO intervention and engaged people living with SMI who may typically be framed within literature as harder to reach with 80% follow‐up. The SW‐CRT design ensured that the intervention was delivered to all service teams, service users and carers and enabled a rigorous evaluation of impacts. Despite clear codesign objectives and evidence that improvements were further codesigned and implemented, there was still a null effect. On the one hand, this may reinforce current debates about the challenges of codesigned service improvements being measured at an individual level.^20^ On the other hand, it might be that the codesigned improvements did not target core components of recovery‐oriented care and therefore could not improve psychosocial recovery. It is possible that the reported 18‐year average of living with SMI played a role. Additionally, it is plausible that the codesign processes played a role in the outcomes; however, the qualitative evaluation feedback gathered from all postgroup meetings suggested that participants found value in feeling heard, being involved in decisions about improvements and working together. Where improvements were noted for codesign, it related to increased representation in codesign groups, for example, where carer numbers were lower and time.

The strengths of this study included that the adapted MH ECO intervention was codeveloped by the state consumer and the carer peak agencies, it was piloted before this trial and delivered by lived‐experience coinvestigators in real‐world settings of MHCSS. This demonstrates the feasibility of delivering a structured codesign model such as the adapted MH ECO model for quality improvement within mental health services with multiple groups and to scale. However, if this intervention were to be adopted for service design and quality improvement in the future, it would be essential to retain the training delivered before codesign due to the inherent need for power discussions and agreements on shared decision‐making approaches in the mental health context. Additionally, the adapted MH ECO model could benefit further from the design‐thinking elements of EBCD being more closely in the foreground. A further limitation of the MH ECO model as implemented may be that the adapted method did not use the film components that the broader EBCD model supports.

A weakness could be that the codesigned improvements did remain mostly quick fixes and process‐oriented. Recent work that has examined the experiences that people might draw from their service encounters is important to consider here as it might point to a need to widen the lens of service users engaged in sharing their stories and to identify further who is taking part in codesign.^36^ There may be a need in EBCD to establish the backgrounds of service users and carers for participation in codesign, so that multiple service encounters or engagement with specific therapeutic models are explicitly sampled for more tangible effects to be realized and sustained. Despite this possible need, the trial did adopt multiple recruitment strategies to enhance representativeness by passive mail‐outs, staff providing a bespoke study postcard to return and express interest and the provision of on‐site recruitment days where people with lived‐experience of mental illness who had been trained in the Stand Up for Mental Health programme delivered by Canadian David Granirer delivered comedy to break‐down silos, address stigma and reduce the burden of participating in formalized research.

In addition to sampling for different service stories, our previously published work outlining an explanatory theoretical model of change for codesign and coproduction in healthcare improvement is highly relevant. In that explanatory model, we noted eight mechanisms of change that are essential for the relational work of EBCD. These mechanisms include recognition (of the importance of experiential evidence and narrative identities), dialogue (to share stories and give equal weight to what is shared and to ensure that conversations do not close off others), cooperation (agreeing to work together in the context of polyphony where there is unlikely to be 100% agreement all of the time), accountability (a shared responsibility for change), mobilisation (generating the movement for change to happen), enactment (making change happen), creativity (using creative approaches and design thinking intentionally for transformation) and attainment (making implementation visible and experiencing those benefits). These mechanisms, we argued, interact with ideal relational transitions that may be observed through codesign processes where individuals transform a position held on issues and experiences in services, and with the people they might be codesigning with. In this ideal, people might be said to move along a relational continuum of being solo and disconnected I's (here, others are typically viewed separately to ‘them’) and through codesign processes, people begin to see others and their vulnerabilities through shared understanding of ‘You’ that forms. This process continues and, longer term, may enable ‘Us’ to form in a way where ‘We’ emerges to enact and attain change. It is possible to suggest, given the null effect that greater attention to the eight mechanisms of change might have fostered relational conditions that could bolster psychosocial recovery. An analysis of this aspect of trial data is currently underway for the process evaluation.

## 6 CONCLUSIONS

There have been more than 60 noncontrolled evaluations of EBCD quality improvement projects^37^ where patient experience and transformations to healthcare workforce, culture, values and behaviours have been documented to improve.^22^ As mental health policy increasingly advocates for coproduction and codesign approaches^37^, ^38^ to facilitate better services, improved experiences and outcomes, there is a need for controlled studies to measure impacts. Using more rigorous SW‐CRT design could assist in this. The broader implications of these findings might best be reflected by consideration of the main action areas identified by people living with SMI and their carers that highlighted the central concerns of better communication, the importance of involvement in decision‐making and the provision of opportunities for being with other people with similar life experiences not solely because of having a diagnosis of mental illnesses. Service providers are challenged daily to truly listen to what people are saying that they want and need and to reflect on their own part and role in providing services that can facilitate hope, meaning and empowerment. The results of this trial confirm the importance of person‐centred care and recovery‐oriented mental health systems, but they show that codesign on its own is inadequate.^39^ The results of this trial may indicate, then, that health policy is to some degree ahead of practice particularly in terms of what we might be able to expect of codesign. Coproduction and codesign in healthcare improvement are essential to the future of mental healthcare reforms, but it is essential that these methods demonstrate how power dynamics and shared decision‐making are attended to.^18^ The critical message for healthcare policy‐makers, service delivery providers and service reformers in this current era of participation in service design and quality improvement is to ask the difficult questions about what outcomes we should expect of coproduction and codesign. We might also require research efforts to elicit what people engaged in codesign expect from it and what kinds of methods and approaches work best for whom, when and under what circumstances. This will require multiple study designs and trials to compare quality improvement methods and, moreover, it is dependent on the engagement of people with lived‐experience of mental illnesses on setting out the expectations of codesign to be able to better determine the effects on service experiences and individual outcomes.

## CONFLICT OF INTERESTS

The authors declare that there are no conflict of interests.

## AUTHOR CONTRIBUTIONS

Victoria J. Palmer and Jane M. Gunn substantially contributed to the study conception, and all the authors contributed to the design. Kali Godbee and Konstancja Densley contributed to the acquisition of data and analysis, and Victoria J. Palmer, Jane M. Gunn, Helen Herrman, John Furler, David Pierce, Patty Chondros and Kali Godbee all contributed to the interpretation of data for the research findings. All authors reviewed drafts of the manuscript and contributed to revised content for publication. All authors accept accountability and responsibility for the content in this publication.

## Supporting information

Supporting information.

Supporting information.

Supporting information.

Supporting information.

Supporting information.

## Data Availability

The data that support the findings of this study are available on request from the corresponding author. The data are not publicly available due to privacy or ethical restrictions.
