# Supplementary material for: The CORE study—An adapted mental health experience codesign intervention to improve psychosocial recovery for people with severe mental illness: A stepped wedge cluster randomized‐controlled trial
Source: Health Expect. 2021 Aug 4;24(6):1948–61. doi: 10.1111/hex.13334 (PMC8628597; doi:10.1111/hex.13334)
Supplement: Supplementary file 1 — Supporting information. [file HEX-24-1948-s002.docx]

Appendix 3

Supplementary Table 3: Estimated within–cluster correlation and within–individual correlation for the primary and secondary outcome (N=287 service users, 841 observations, 9 clusters)

| **Outcomes** | **Within–cluster correlation**^1^ | **Within–individual**  **correlation**^1^ |
| --- | --- | --- |
| **RAS–R total** | 0·02 | 0·73 |
| **RAS–R sub–domains** |  |  |
| Personal Confidence and Hope | 0·02 | 0·73 |
| Willingness to Ask for Help | 0·001 | 0·57 |
| Goal and Success Orientation | 0·009 | 0·65 |
| Reliance on Others | 0·02 | 0·55 |
| Not Dominated by Symptoms | 0·03 | 0·51 |
| **EUROHIS_8 QoL TOTAL** | 0·03 | 0·70 |

^1^ Estimated using the linear mixed effects model to compare the intervention and control phases, adjusted for follow–up time point.
