# Supplementary material for: The CORE study—An adapted mental health experience codesign intervention to improve psychosocial recovery for people with severe mental illness: A stepped wedge cluster randomized‐controlled trial
Source: Health Expect. 2021 Aug 4;24(6):1948–61. doi: 10.1111/hex.13334 (PMC8628597; doi:10.1111/hex.13334)
Supplement: Supplementary file 2 — Supporting information. [file HEX-24-1948-s004.docx]

Appendix 2

Supplementary Table 2: Summary statistics of primary and secondary outcomes by intervention and control phase, overall and for each follow–up time point for people with severe mental illness (N=287, 841 observations)

|  |  | **Follow–up time point** | | | |
| --- | --- | --- | --- | --- | --- |
|  | **Overall**  **(n=287)** | **Baseline**  **(n=235)** | **9 months**  **(n=229)** | **18 months**  **(n=198)** | **27 months (n=179)** |
| **Intervention phase compared to control phase at each follow–up time point** | | | | | |
|  | **Number of observations** | **n** | **n** | **n** | **n** |
| Intervention phase | 375 | – | 67 | 129 | 179 |
| Control phase | 466 | 235 | 162 | 69 | – |
|  | **Mean (SD)** | **Mean (SD)** | **Mean (SD)** | **Mean (SD)** | **Mean (SD)** |
| **RAS–R (Range 24 to 120)** |  |  |  |  |  |
| Intervention phase | 84·7 (15·6) | – | 80·5 (15·7) | 86·5 (15·3) | 85·1 (15·6) |
| Control phase | 86·5 (15·3) | 87·1 (15·3) | 86·3 (15·0) | 85·1 (15·8) | – |
|  |  |  |  |  |  |
| **Personal Confidence and Hope (Range 9 to 45)** | |  |  |  |  |
| Intervention phase | 30·3 (6·9) | – | 28·5 (7·2) | 30·8 (6·9) | 30·6 (6·8) |
| Control phase | 30·9 (6·7) | 31·0 (6·8) | 31·0 (6·5) | 30·3 (6·7) | – |
|  |  |  |  |  |  |
| **Willingness to Ask for Help (Range 5 to 25)** | |  |  |  |  |
| Intervention phase | 11·4 (3·0) | – | 10·7 (3·5) | 11·6 (2·7) | 11·4 (2·9) |
| Control phase | 11·7 (2·7) | 11·6 (2·7) | 11·8 (2·5) | 11·6 (2·7) | – |
|  |  |  |  |  |  |
| **Goal and Success Orientation (Range 3 to 15)** | |  |  |  |  |
| Intervention phase | 18·4 (4·1) | – | 17·6 (4·1) | 18·9 (4·0) | 18·3 (4·1) |
| Control phase | 19·1 (3·8) | 19·3 (3·7) | 19·0 (3·7) | 18·6 (4·1) | – |
|  |  |  |  |  |  |
| **Reliance on Others (Range 4 to 25)** | |  |  |  |  |
| Intervention phase | 15·6 (2·6) | – | 15·6 (2·5) | 15·6 (2·5) | 15·6 (2·7) |
| Control phase | 15·7 (2·9) | 15·8 (2·9) | 15·4 (2·9) | 15·8 (2·5) | – |
|  |  |  |  |  |  |
| **Not Dominated by Symptoms (Range 3 to 15)** | |  |  |  |  |
| Intervention phase | 9·1 (3·0) | – | 8·1 (3·2) | 9·6 (2·8) | 9·1 (2·9) |
| Control phase | 9·3 (3·0) | 9·4 (3·0) | 9·2 (2·9) | 8·8 (3·0) | – |
|  |  |  |  |  |  |
| **EUROHIS–8 QoL (Range 8 to 40)** | |  |  |  |  |
| Intervention phase | 24·6 (6·7) | – | 22·5 (6·7) | 25·1 (6·6) | 25·1 (6·6) |
| Control phase | 25·4 (6·5) | 25·5 (6·6) | 25·7 (6·5) | 24·1 (5·9) | – |
|  |  |  |  |  |  |
| **Length of time exposed to the intervention at each follow–up time point** | | | | | |
|  | **Number of observations** | **n** | **n** | **n** | **n** |
| 0 months | 466 | 235 | 162 | 69 | – |
| 9 months | 200 | – | 67 | 71 | 62 |
| 18 months | 124 | – | – | 58 | 66 |
| 27 months | 51 | – | – | – | 51 |
|  | **Mean (SD)** | **Mean (SD)** | **Mean (SD)** | **Mean (SD)** | **Mean (SD)** |
| **RAS–R (Range 24 to 120)** |  |  |  |  |  |
| 0 months | 86·5 (15·3) | 87·1 (15·3) | 86·3 (15·0) | 85·1 (15·8) | – |
| 9 months | 84·7 (15·3) | – | 80·5 (15·7) | 89·2 (14·6) | 84·1 (14·4) |
| 18 months | 85·8 (15·5) | – | – | 83·2 (15·5) | 88·2 (15·2) |
| 27 months | 82·2 (17·0) | – | – | – | 82·2 (17·0) |
|  |  |  |  |  |  |
| **Personal Confidence and Hope (Range 9 to 45)** | |  |  |  |  |
| 0 months | 30·9 (6·7) | 31·0 (6·8) | 31·0 (6·5) | 30·3 (6·7) | – |
| 9 months | 30·4 (6·9) | – | 28·5 (7·2) | 32·3 (6·4) | 30·5 (6·7) |
| 18 months | 30·6 (6·9) | – | – | 29·0 (7·1) | 32·0 (6·4) |
| 27 months | 28·9 (7·1) | – | – | – | 28·9 (7·1) |
|  |  |  |  |  |  |
| **Willingness to Ask for Help (Range 5 to 25)** | |  |  |  |  |
| 0 months | 11·7 (2·7) | 11·6 (2·7) | 11·8 (2·5) | 11·6 (2·7) | – |
| 9 months | 11·4 (2·9) | – | 10·7 (3·5) | 12·0 (2·6) | 11·4 (2·5) |
| 18 months | 11·5 (2·7) | – | – | 11·2 (2·8) | 11·7 (2·7) |
| 27 months | 11·1 (3·5) | – | – | – | 11·1 (3·5) |
|  |  |  |  |  |  |
| **Goal and Success Orientation** | |  |  |  |  |
| 0 months | 19·1 (3·8) | 19·3 (3·7) | 19·0 (3·7) | 18·6 (4·1) | – |
| 9 months | 18·5 (4·0) | – | 17·6 (4·1) | 19·5 (3·8) | 18·4 (4·0) |
| 18 months | 18·6 (3·9) | – | – | 18·2 (4·2) | 19·0 (3·7) |
| 27 months | 17·5 (4·6) | – | – | – | 17·5 (4·6) |
|  |  |  |  |  |  |
| **Reliance on Others (Range 4 to 25)** | |  |  |  |  |
| 0 months | 15·7 (2·9) | 15·8 (2·9) | 15·4 (2·9) | 15·8 (2·5) | – |
| 9 months | 15·5 (2·5) | – | 15·6 (2·5) | 15·5 (2·7) | 15·4 (2·4) |
| 18 months | 15·6 (2·8) | – | – | 15·6 (2·4) | 15·7 (3·1) |
| 27 months | 15·8 (2·6) | – | – | – | 15·8 (2·6) |
|  |  |  |  |  |  |
| **Not Dominated by Symptoms (Range 3 to 15)** | |  |  |  |  |
| 0 months | 9·3 (3·0) | 9·4 (3·0) | 9·2 (2·9) | 8·8 (3·0) | – |
| 9 months | 8·9 (2·9) | – | 8·1 (3·2) | 10·0 (2·6) | 8·5 (2·7) |
| 18 months | 9·5 (2·9) | – | – | 9·2 (3·0) | 9·8 (2·9) |
| 27 months | 8·9 (3·2) | – | – | – | 8·9 (3·2) |
|  |  |  |  |  |  |
| **EUROHIS–8 QoL (Range 8 to 40)** | |  |  |  |  |
| 0 months | 25·4 (6·5) | 25·5 (6·6) | 25·7 (6·5) | 24·1 (5·9) | – |
| 9 months | 24·7 (6·3) | – | 22·5 (6·7) | 26·8 (6·0) | 24·6 (5·5) |
| 18 months | 24·8 (7·2) | – | – | 23·0 (6·8) | 26·3 (7·3) |
| 27 months | 24·0 (6·6) | – | – | – | 24·0 (6·6) |

n - number of individuals who responded; SD - standard deviation
