# Supplementary material for: The CORE study—An adapted mental health experience codesign intervention to improve psychosocial recovery for people with severe mental illness: A stepped wedge cluster randomized‐controlled trial
Source: Health Expect. 2021 Aug 4;24(6):1948–61. doi: 10.1111/hex.13334 (PMC8628597; doi:10.1111/hex.13334)
Supplement: Supplementary file 3 — Supporting information. [file HEX-24-1948-s005.pdf]

THE PRIORITISATION OF TOUCH POINTS ACROSS GROUPS TO IDENTIFY THE CO-DESIGN OBJECTIVES USING EMOTION MAPPING

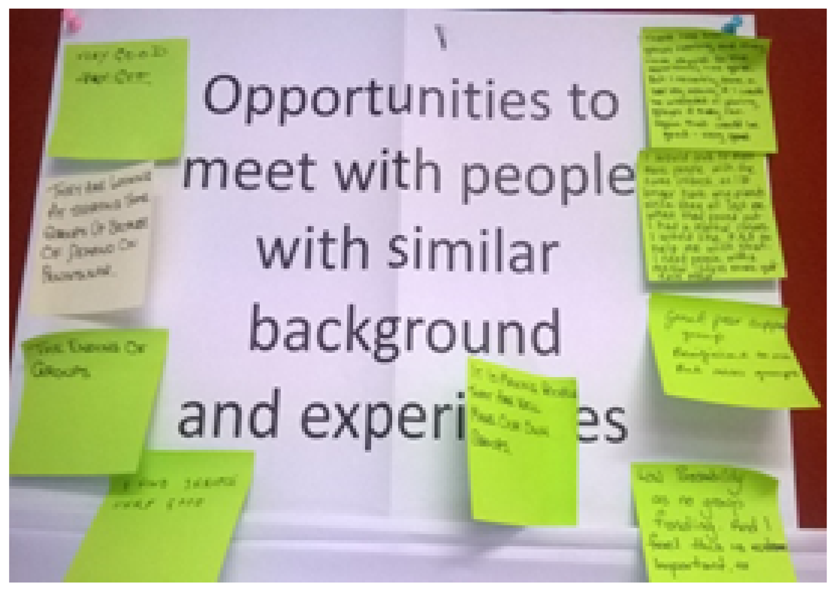

Very good. The ending of groups is making people that are well make our own groups. Low possibility as no group funding and I feel this is important too. Great peer support group, beneficial to me but miss groups,. I want to meet people with the same interest, to get to know each other. The opportunity has gone since they stopped the groups

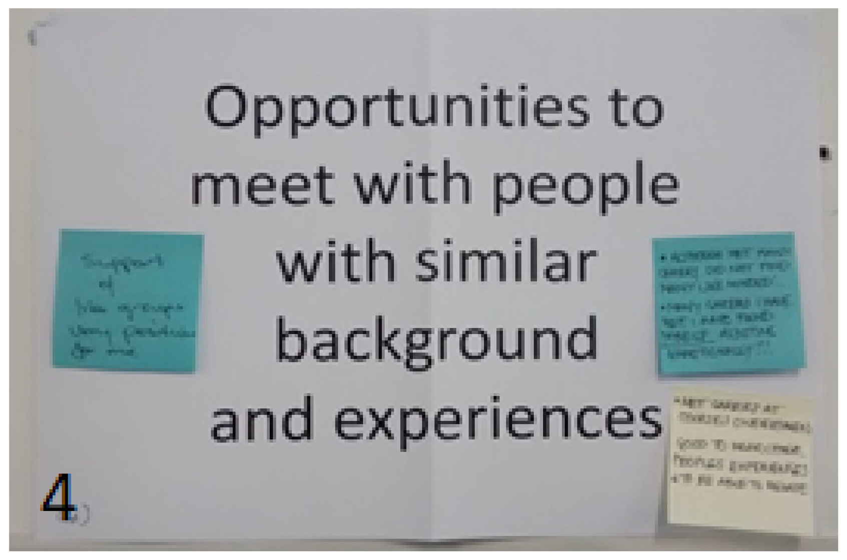

Support of like groups very positive for me, although have not met many 'like minded', many carers I have met I have myself assisting emotionally, met carers at courses undertaken, good to hear other peoples experiences to be able to relate.

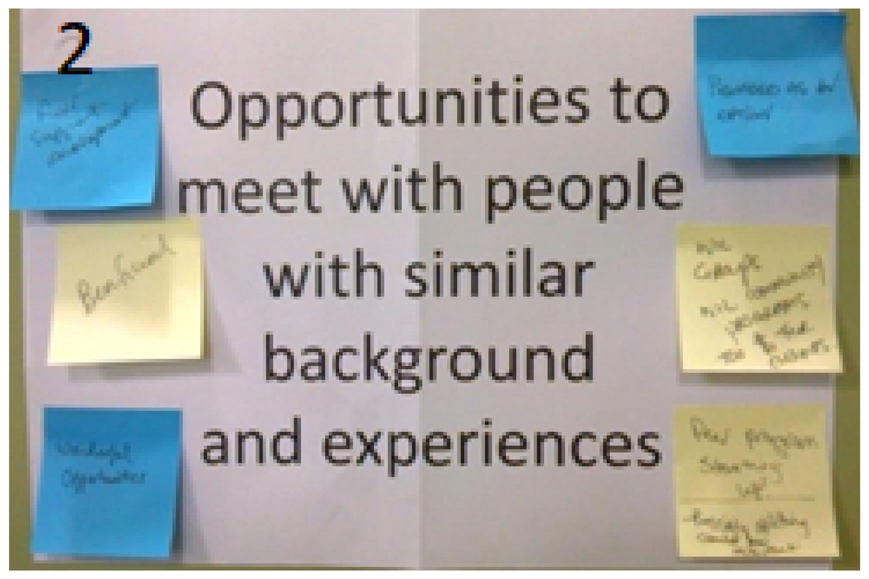

Peer support development, wonderful opportunities, provided as an option, beneficial, peer program starting up. Nil groups, nil community programs, too expensive for clients.

CO-DESIGN OBJECTIVES

1) TO DEVELOP A PLAN TO IMPROVE OPPORTUNITIES TO MEET WITH PEOPLE WITH SIMILAR BACKGROUNDS AND EXPERIENCES

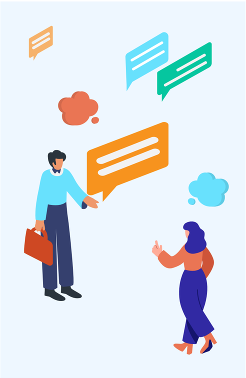

(2) TO DEVELOP A PLAN TO IMPROVE COMMUNICATION OF INFORMATION TO SERVICE USERS, CARERS, STAFF AND THE WIDER COMMUNITY

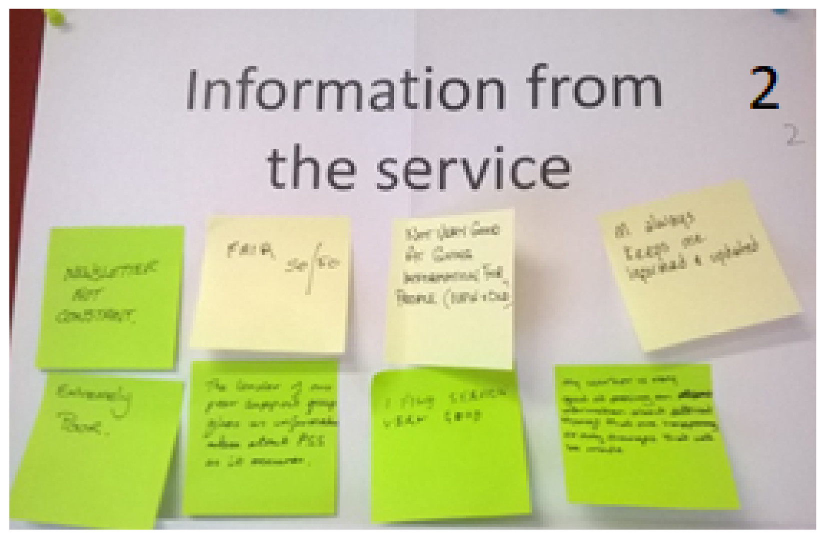

Newsletter not constant, extremely poor, leader of our peer support group give us information about service as it occurs, very good, worker always keeps me informed and updated. Not very good at giving information for people (new and old). Fair 50/50.

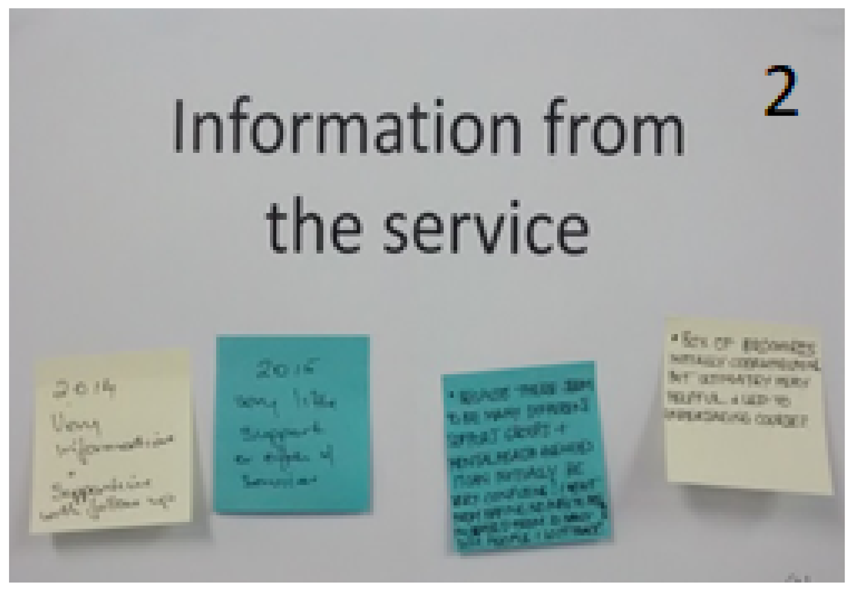

2015 Very little support or offer of services, there seem to be many different support groups and mental health agencies it can initially be very confusing, I went from having no info to being inundated from so many different people I lost track! 2014 Very informative, supportive with follow up, box of brochures initially overwhelming but ultimately very helpful and led to undertaking courses

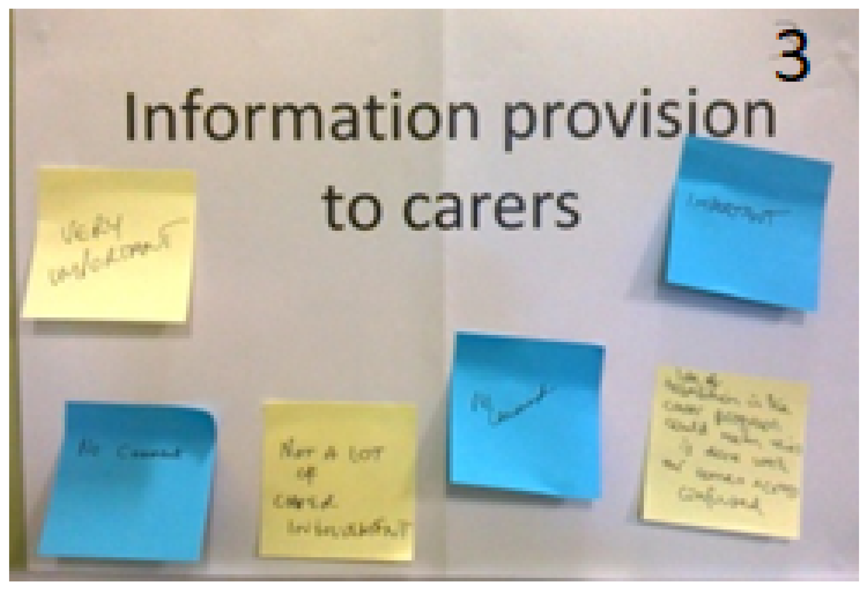

Very important, lots of transition in the carer program, could mean this is done well or comes across confused. not a lot of carer involvement, no comment.

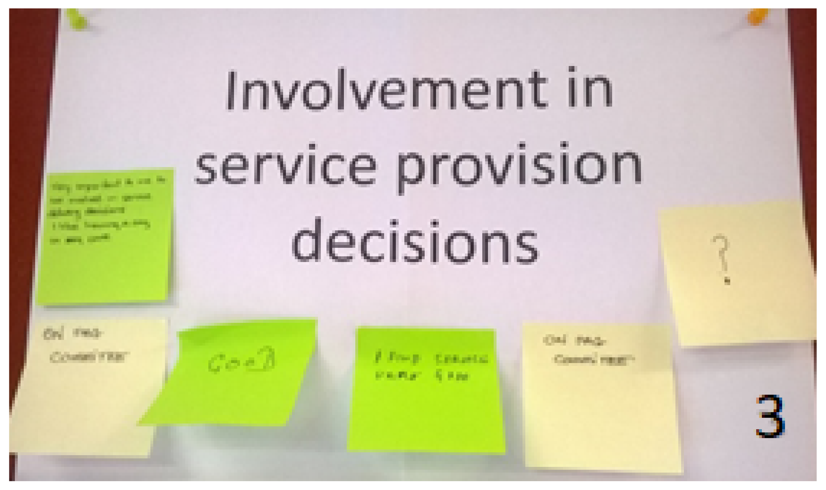

Very good. Very important to me to be involved in service delivery decisions. I like having a say in my care. On peer advisory committee

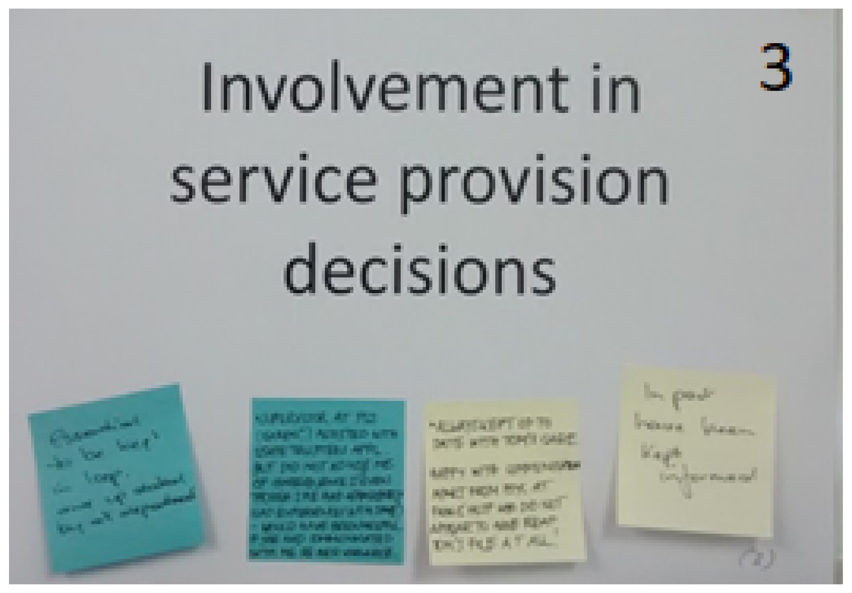

Essential to be kept in the loop, supervisor at service assisted with state trustees application but did not let me know of consequences, would have been helpful if she had communicated with me re her knowledge, in the past have been kept informed. Always kept up to date with person's care, happy with communication apart from psych at hospital who did not appear to have read person's file at all.

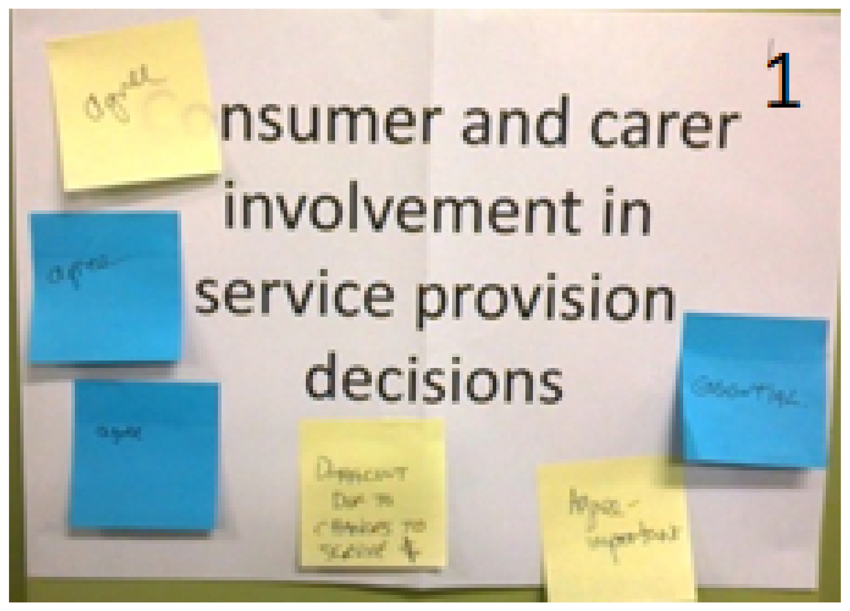

Agree, important, essential. difficult due to changes in services

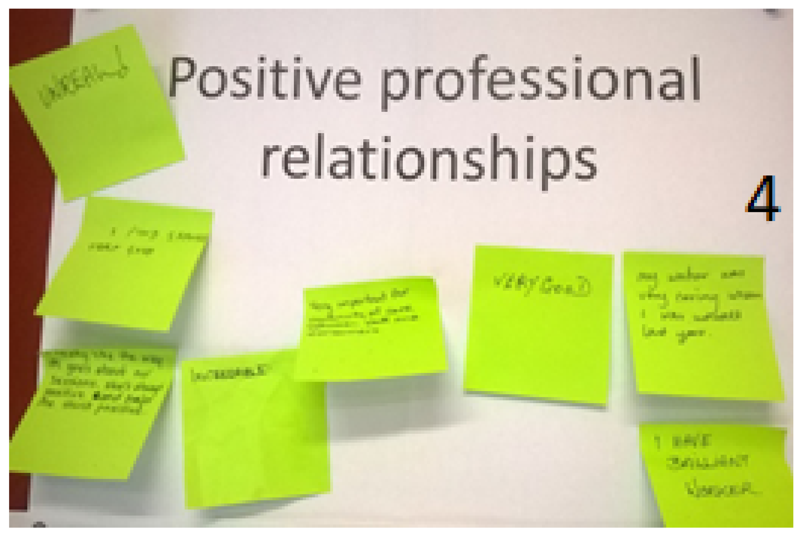

Unreal!. I find services very good. I really like the way worker goes about our sessions. They're always positive and help me think positively. Incredible. Very important for continuity of care between staff and consumers. My worker was very caring when I was unwell last year, I have a brilliant worker.

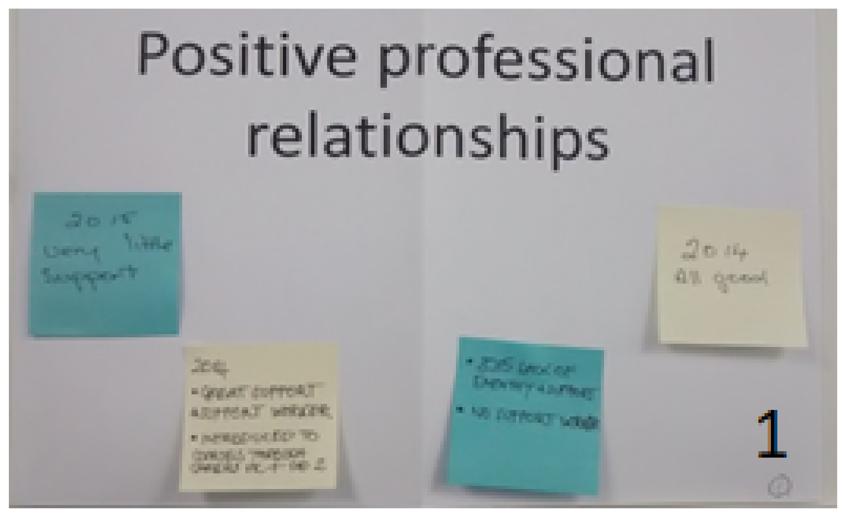

2015 Very little support and lack of empathy. No support worker. 2014 All good, great support and support worker. Introduced to courses through Carers VIC and did two.

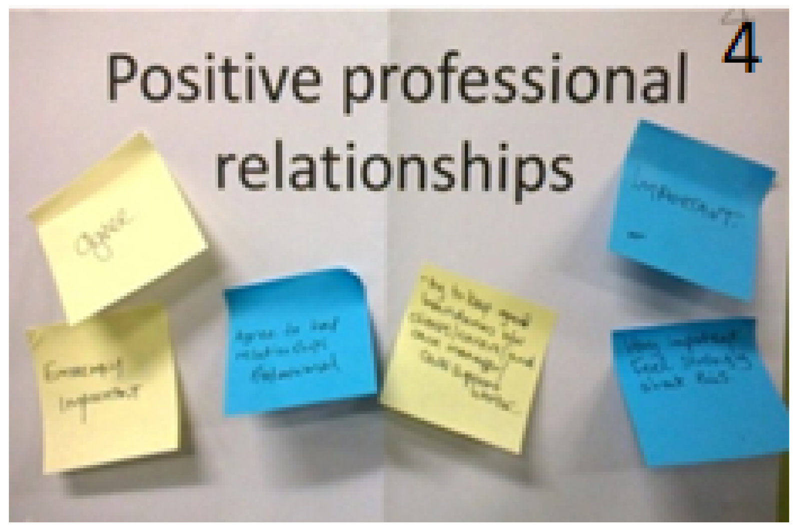

Agree to keep relationships professional, very and extremely important . Feel strongly about this. Try to keep good boundaries between clients/carer/case manager/carer support worker.
