# Supplementary material for: The CORE study—An adapted mental health experience codesign intervention to improve psychosocial recovery for people with severe mental illness: A stepped wedge cluster randomized‐controlled trial
Source: Health Expect. 2021 Aug 4;24(6):1948–61. doi: 10.1111/hex.13334 (PMC8628597; doi:10.1111/hex.13334)
Supplement: Supplementary file 4 — Supporting information. [file HEX-24-1948-s001.docx]

Appendix 1

Supplementary Table 1: Missing data patterns across the follow–up time points for people with severe mental illness (N=287)

| **Follow–up time point** | | | | |
| --- | --- | --- | --- | --- |
| **Baseline** | **9 months** | **18 months** | **27 months** | **n** |
| **Entered study at baseline (n=235)** | | | | |
| 1 | 1 | 1 | 1 | 130 |
| 1 | 1 | 1 | 0 | 20 |
| 1 | 1 | 0 | 1 | 7 |
| 1 | 0 | 1 | 1 | 7 |
| 1 | 1 | 0 | 0 | 35 |
| 1 | 0 | 1 | 0 | 1 |
| 1 | 0 | 0 | 1 | 1 |
| 1 | 0 | 0 | 0 | 34 |
| **Entered study at 9 months (n=37)** | | | | |
| –– | 1 | 1 | 1 | 22 |
| –– | 1 | 1 | 0 | 5 |
| –– | 1 | 0 | 1 | 1 |
| –– | 1 | 0 | 0 | 9 |
| **Entered study at 18 months (n=15)** | | | | |
| –– | –– | 1 | 1 | 9 |
| –– | –– | 1 | 0 | 4 |
| **Entered study at 27 months (n=2)** | | | | |
| –– | –– | –– | 1 | 2 |
