# Supplementary material for: The CORE study—An adapted mental health experience codesign intervention to improve psychosocial recovery for people with severe mental illness: A stepped wedge cluster randomized‐controlled trial
Source: Health Expect. 2021 Aug 4;24(6):1948–61. doi: 10.1111/hex.13334 (PMC8628597; doi:10.1111/hex.13334)
Supplement: Supplementary file 5 — Supporting information. [file HEX-24-1948-s003.docx]

Appendix Two: The CORE Study Feedback summary from all participant groups for co-design training sessions

| **What did you hope to get out of today’s meeting?** | **What worked well in today’s meeting?** | **What could have gone better in today’s meeting?** |
| --- | --- | --- |
| - To hear and learn more about CORE and understand its expectations and the next steps and understanding my purpose in involvement - To learn and participate more actively in a group setting, be heard and mix with different people - Understand, obtain clearer knowledge of the process and the role of each group and how it operates and improve group relations - Obtain a clearer and better understanding of co-design, its importance and, the way it works and have an idea of how it can improve the service - Information to help me go on to have more confidence and what part I play and what role may be as well as meeting other members feeling comfortable enough to be able to contribute to the group - To get a sense of engagement and to help design and implement mental health services in Victoria - No expectations, here just to help | - Communication and active listening between each other where everyone had equal opportunities to speak and be respectful of each other’s thoughts feeling part of the group where people had open and reflective discussions and given a chance to voice their opinion - Feeling comfortable and confident, included and meeting other people with similar problems and experiences and that a lot of our concerns are the same - Clarifying and explaining the process by using the video and slides, encouraging to see what benefits could come from this type of study - Role playing in understanding the structure, group work and discussion well explained and facilitated which worked really well, “I usually don’t like role play but I found this very interesting and doing the actual activity which was a good example” - The folder and examples provided were great with information is to read and follow when referred to - Small group setting which was relaxed with friendly staff where the penny dropped, group work is effective - Loved the structure and inclusiveness of the process, it ran smoothly and all were able to contribute | - Participants punctuality - More practical activities or discussions, more time for the activities and more keeping on task - At times people interrupting, one person talking at a time - Some language too technical and information repetitive but also too slow paced/easy - Maybe the introduction could be more concise, overview and orientation of and using the folder, page numbers, wasn’t aware a copy of slides were in the folder - Quieter space for group discussion (have two rooms) and room temperature - Audio visual and technology - More info on what research project hopes to impact in real context of mental health services |

The CORE Study Feedback summary from all co-design meeting participant groups

| **What did you hope to get out of today’s meeting?** | **What worked well in today’s meeting?** | **What could have gone better in today’s meeting?** |
| --- | --- | --- |
| - Clarity and understanding of the co-design process and its importance, the action plan and the objective - Positive outcomes and assisting in improvements to the service and how mental health organisations function; how to improve services based on clients/carers feedback - To make a change in mental health support and obtain a greater understanding of new developments in mental health service provision - Contribute to the process to help other service users, reach goal of deciding on objective and implementing change and working further on formalising specific areas and tasks for improvement and strategies - An understanding of experiences and perspectives of service users, carers and staff and involvement of all in decision making process and how best to provide a consumer sensitive service; plan for quality improvement identified by service users and carers - A better understanding of the services process and how it operates, who they are, what are their services and implementing new processes - Group development, a clearer understanding of groups, how information is disclosed within groups and working more effectively as a team and being an active participant in the discussions - Sharing of experiences with others to accommodate people with disability and MH experiences and understanding constraints of workers | - Group participation, everyone was given equal opportunity to contribute and respect was shown listening to each others views, cooperative process, good and open discussions that were constructive, productive and educational with great ideas and brainstorming where communication was effective - Preparation of what is to be achieved in the meeting, getting a plan developed and the discussion process - Expanding points of concerns for all and communication, brainstorming the process and developing realistic goals - Management in keeping conversation contained to exploring points to be discussed at subsequent meetings, having a clear agenda and strong chair, effective time management and focussed and thorough discussion - Excellent and effective facilitation and guidance/great presenters, good sharing of time, clear direction and facilitation, cohesive and well led, user friendly process and supportive with emotional support provided to overwhelmed participants - Interesting to hear staff attitude and honesty, pressures and point of views and learnt more about the service | - Putting ideas together more clearly after discussions as some parts of the discussion seemed a bit rushed - Focus direction by chairperson, waiting to contribute and being ignored, some people interrupting so facilitators should contain this - Limited time for people’s personal experiences, more time/pressed for time and opportunity to speak meant difficulty of keeping on track and discussing all areas planned and the group dynamics - A larger group for broader ideas, more involvement of service users and carers and more people attending - A more detailed report of feedback from experiences interviews before meeting would have been of benefit to have time to put more thought and time into the topics - Starting on time, all attendees present and on time and committing to the entire meeting, some arriving late, leaving early) - *It should be a full day conference* - *More cheese!* - *Nothing, all good, well run, good constructive meeting, thank you* |
